# Study on the Evolution Mechanism of Carbon Impurities in Polysilicon Production Based on HSC Simulation

**DOI:** 10.3390/ma19040798

**Published:** 2026-02-18

**Authors:** Yu Hou, Xueqian Lv, Guoqiang Huang

**Affiliations:** 1 School of Chemical Engineering and Technology, Tianjin University, Tianjin 300350, China; 2 Xinjiang Xinte Crystalline Silicon High-Tech Co., Ltd., Urumqi 830011, China; lvxueqian@163.com; 3 State Key Laboratory of Chemical Engineering and Low-Carbon Technology, Tianjin University, Tianjin 300350, China

**Keywords:** polysilicon, carbon impurities, existing forms, evolution mechanism, HSC simulation calculations

## Abstract

The existing forms and evolution mechanisms of carbon impurities constitute the core scientific issue in the optimization of polysilicon purification processes. The depth of research on this issue directly determines the targeting and effectiveness of directional impurity removal strategies, and is even a key prerequisite for improving the quality and reducing the cost of polysilicon products. Based on HSC simulation calculations and using the Gibbs free energy of reactions as the judgment criterion, this paper investigated the existing forms and evolution mechanism of carbon impurities during the production of polysilicon via the modified Siemens process. The results show that the evolution mechanism of carbon impurities is as follows: the solute carbon in silicon powder reacts with hydrogen to generate CH_4_. Subsequently, CH_4_ synergistically undergoes radical rearrangement and the Rochow reaction with methylchlorosilanes in chlorosilane and CH_4_ in recovered hydrogen. Meanwhile, CH_3_· radicals combine with radicals generated from chlorosilanes to form a mixture of methylchlorosilanes dominated by SiH(CH_3_)Cl_2_ as well as CH_4_. After distillation purification, SiH(CH_3_)Cl_2_ enters the SiHCl_3_ stream, and then synergistically undergoes cracking and radical rearrangement with CH_4_ in high-purity hydrogen, the solid-soluble elemental carbon forms and deposits in polysilicon. Simultaneously, a mixture of methylchlorosilanes dominated by SiH(CH_3_)Cl_2_ along with CH_4_ is generated and then fed into the tail gas system. This will provide the necessary theoretical foundation for the development of efficient and low-cost impurity removal strategies.

## 1. Introduction

The growing global demand for energy, coupled with the increasing severity of the greenhouse effect, has accelerated the formation of a new global energy system and facilitated the transition of the global energy system toward low carbonization. New types of green energy, represented by wind power and photovoltaic energy, are poised to embrace new historical development opportunities. High-purity polysilicon is a critical foundational material for the photovoltaic and semiconductor industries, and it is of great significance to the green and high-quality development of the global energy and information sectors. It is mainly produced via the silane fluidized bed process and the improved Siemens method [1,2,3,4]. During the formation of single crystal silicon, carbon impurities in polysilicon will accelerate the formation of oxygen precipitates, thereby inducing secondary defects such as dislocations and stacking faults, affecting the minority carrier lifetime and the lattice arrangement of monocrystalline silicon. These impurities are one of the key factors restricting the photoelectric conversion efficiency of solar cells [1,3,5].

Constructing low-cost and highly selective targeted removal strategies to replace the “one-size-fits-all” broad-spectrum removal mode adopted by traditional impurity removal processes, thus effectively regulating carbon impurities at different stages, is of great significance for breaking through the purity bottleneck of polysilicon and reducing the energy consumption and costs of purification. Systematic clarification of the existing forms and evolution mechanisms of carbon impurities is a necessary foundation for the construction of highly selective targeted strategies. Researchers have conducted extensive studies focusing on the sources, detection and analysis methods, and physical property parameters of carbon impurity compounds, thus yielding a series of innovative achievements. For example, Yuan [6], in summarizing the sources of impurities during the preparation of electronic-grade polysilicon from industrial silicon, proposed control strategies from the aspects of raw material preparation, purification, impurity inhibition mechanisms in the trichlorosilane (SiHCl_3_) separation process, and tail gas separation technologies, and concluded that impurities in high-purity polysilicon are mainly derived from recycled hydrogen, SiHCl_3_ (metallic silicon), graphite electrodes, equipment pipelines, gaskets, and the environment. Based on the analysis results of carbon impurity sources, transformation, and raw material purification, Cao et al. [7] proposed that the carbon impurity concentration in high-purity SiHCl_3_ can be reduced by controlling the carbon impurity concentration in metallurgical silicon and applying the optimal purification technology; meanwhile, the purity of polysilicon can be improved through the coordinated control of the methane (CH_4_) concentration in recovered hydrogen. Chen [8] employed gas chromatography–mass spectrometry (GC-MS) to analyze the speciation of polychlorosilanes and methylchlorosilanes in synthetic chlorosilanes (produced via direct chlorination), and concluded that carbon impurities in synthetic chlorosilanes are primarily composed of monomethyldichlorosilane (SiH(CH_3_)Cl_2_), dimethyldichlorosilane (Si(CH_3_)_2_Cl_2_), and methyltrichlorosilane (Si(CH_3_)Cl_3_). Yang [9] established a rapid determination method (GC-MS) for SiH(CH_3_)Cl_2_, trimethylchlorosilane (Si(CH_3_)_3_Cl), Si(CH_3_)Cl_3_, and Si(CH_3_)_2_Cl_2_ in high-purity SiHCl_3_ using 1,2-dichloroethane as the internal standard. The detection limit and reproducibility of this method can meet the testing requirements for carbon impurities in SiHCl_3_ used in polysilicon production, and it has also become a universally adopted method in the industry at present. Lewis et al. [10] reported gas chromatography and infrared spectroscopy analytical methods related to carbon impurities in chlorosilanes. Coleman et al. [11] identified the possible existing forms of impurities in chlorosilanes and separately provided key property parameters of the relevant compounds, such as saturated vapor pressure equations, boiling points, critical temperatures, critical pressures, and critical volumes, aiming to provide a basis for the simulation calculation of rectification columns and their operating parameters. Vaisarová [12] provided the dipole moments of chlorine-containing organosilicon compounds, which will serve as a basis for the selection of adsorbents. In addition to the aforementioned studies, research on carbon impurities also covers aspects such as the transformation of carbon-containing compounds in SiHCl_3_ [10,13,14,15,16], and the impact of CH_4_ content in hydrogen on the quality of polysilicon [6]. However, the content of carbon impurities in raw materials is at a trace level, affected by the detection limits of testing equipment and the stability of compounds, so the accurate characterization of the existing forms and evolution processes of carbon impurities at different stages is limited, and existing detection technologies cannot achieve the accurate detection of all compounds. Owing to the influence of the reaction conditions of the Si-H-Cl system, it is extremely challenging to achieve the online detection of all carbon impurity-related elements and compounds. No hydrocarbon compounds such as ethane, CO and CO_2_, have been detected in hydrogen; and no tetramethylsilane, monomethylmonochlorosilane and other such substances have been detected in chlorosilanes. However, it is necessary to further clarify their presence, given that these substances exert a significant impact on intermediate products and high-purity polysilicon.

Based on the above understanding, this paper starts from the key processes of polysilicon production (direct chlorination, cold hydrogenation, and reduction reaction), combines thermodynamic calculation and experimental characterization methods, and analyzes the dominant existing forms and evolution driving forces of carbon impurities at each stage, as well as the evolution rules of carbon impurities during polysilicon production. This will provide a theoretical basis for the development of phased targeted impurity removal strategies and the improvement in polysilicon quality and reduction in cost.

## 2. Materials and Methods

### 2.1. Thermodynamic Calculation Modeling Methods

Gibbs free energy is a crucial thermodynamic function, typically employed to determine the direction and extent of a reaction under specific temperature and pressure conditions. Generally, the lower the Gibbs free energy of a reaction, the greater the driving force of the spontaneous reaction, and the more spontaneously the reaction proceeds [17].

HSC Chemistry (version: HSC 6.0) is a simulation software based on fundamental concepts of chemical thermodynamics, designed to calculate thermodynamic parameters of substances under ideal conditions. The commonly used functions of HSC Chemistry include the calculation of thermodynamic parameters for chemical reactions, heat and material balance calculations, electrochemical equilibrium calculations, and Pourbaix diagram plotting, among others. A key advantage of this software is that it eliminates the need to consider the kinetics of chemical reactions and the non-ideality of solutions, enabling the efficient and convenient verification of the thermodynamic validity of reaction equations involving arbitrary reactants and products, along with the calculation of their corresponding thermodynamic parameters. Thus, it is typically applied in the calculation of thermodynamic parameters for chemical reactions, heat and material balance calculations, etc. In accordance with the laws of thermodynamics, the Gibbs free energy of a reaction is closely correlated with the equilibrium constant and the equilibrium composition of species. In this work, the reaction equation module of the HSC simulation software (HSC 6.0, Metso Finland Oy, Espoo, Finland) was employed to compute the Gibbs free energy of the relevant reactions [18], so as to predict the tendency of formation of the corresponding reaction products.

### 2.2. Materials and Experiments

The metallurgical silicon (≥99%), polysilicon (≥99.99999999), SiHCl_3_, silicon tetrachloride (SiCl_4_), and recycled hydrogen (≥99.9%), were supplied by Xinjiang Xinte Crystalline Silicon High-Tech Co., Ltd. (Urumqi, China). The carbon content in metallurgical silicon was determined using the infrared absorption method (carbon-sulfur analyzer, SC-2800, NCS Testing Technology Co., Ltd., Beijing, China) [19], high-frequency heating and pure oxygen combustion were employed, with a sample mass of 1.5 g per test run and three replicate samples prepared for each sample. The carbon impurity contents in SiHCl_3_ and SiCl_4_ were measured via chromatography–mass spectrometry (GC-MS, 8850-5977, Agilent Technologies, Inc., Santa Clara, CA, USA) [20], the type of column used in this work was DB-VRX, the carrier gas was He; for the heating program, the initial temperature was set at 40 °C and held for 16 min, then ramped up to 200 °C at a rate of 50 °C/min and held for 1 min for detection. The actual value was determined as the average of the initial test values obtained from three replicate samples. For recycled hydrogen, the carbon impurity content was analyzed by gas chromatography (GC, SC-8100, Chongqing Chuanyi Automation Co., Ltd., Chongqing, China) [21]. The phase compositions of metallurgical silicon and polysilicon were characterized using an X-ray diffractometer (XRD, Smart Lab 9 kW, Rigaku Corporation, Hokuto City, Yamanashi Prefecture, Japan) [22], using CuKα radiation at a scan rate of 2°/min. In this study, Bragg–Brentano diffraction geometry was adopted to achieve a mechanical coupling motion mode with a 1:2 angular ratio of θ to 2θ. The variation curve of diffraction intensity as a function of the 2θ angle was automatically captured and recorded, thereby generating the corresponding X-ray diffraction (XRD) patterns. For silicon powder and polycrystalline silicon, two replicate samples were tested for each material, and the results obtained were almost identical. Therefore, one representative diffraction pattern from each was selected for presentation in this paper, and the phase composition was analyzed on the basis of the standard single-phase PDF cards. Furthermore, due to the low carbon impurity content in polysilicon, polysilicon was fabricated into single crystal ingots via the floating zone (FZ) melting technique in accordance with the national standard GB/T 29057 [23]. Subsequently, the carbon content was measured using a cryogenic Fourier transform infrared spectrometer (CryoSAS, Bruker Corporation, Ettlingen, Germany) [24].

## 3. Results and Discussion

### 3.1. Technological Route for Polysilicon Preparation via the Modified Siemens Process

**Figure 1:**
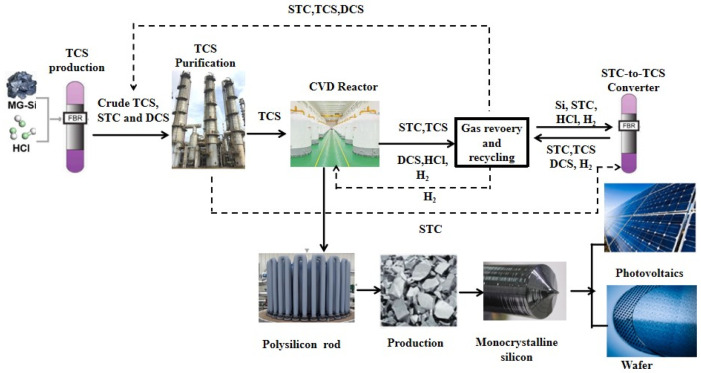
Technological route for polysilicon preparation via the modified Siemens process and its applications.

The technology route of the modified Siemens process is shown in Figure 1. Under specific temperature (540–560 °C) and pressure (2.5–3.5 MPa) conditions, metallurgical silicon, hydrogen, and SiCl_4_ undergo a chemical reaction to generate a chlorosilane mixture (via cold hydrogenation, with the remainder supplemented by the reaction of metallurgical silicon and hydrogen chloride (HCl, direct chlorination, 280~320 °C, 0.25~0.30 MPa). Subsequently, a series of purification and separation steps are conducted to obtain high-purity refined SiHCl_3_ (distillation process). The refined SiHCl_3_ is then fed into a reduction furnace (reduction process), where it undergoes a chemical vapor deposition (CVD, 1000~1100 °C, 0.6 MPa) reaction with high-purity hydrogen to produce polysilicon. Meanwhile, a comprehensive tail gas recovery system is equipped to ensure the full utilization of materials (tail gas recovery process), thereby achieving the closed-loop production of polysilicon. The main introduction points of carbon impurities in polysilicon are cold hydrogenation (direct chlorination), CVD, and tail gas recovery, with the primary material sources being metallurgical silicon, SiCl_4_, SiHCl_3_, and recovered hydrogen.

### 3.2. Existence Forms of Carbon Impurities in Metallurgical Silicon and Polysilicon

**Figure 2:**
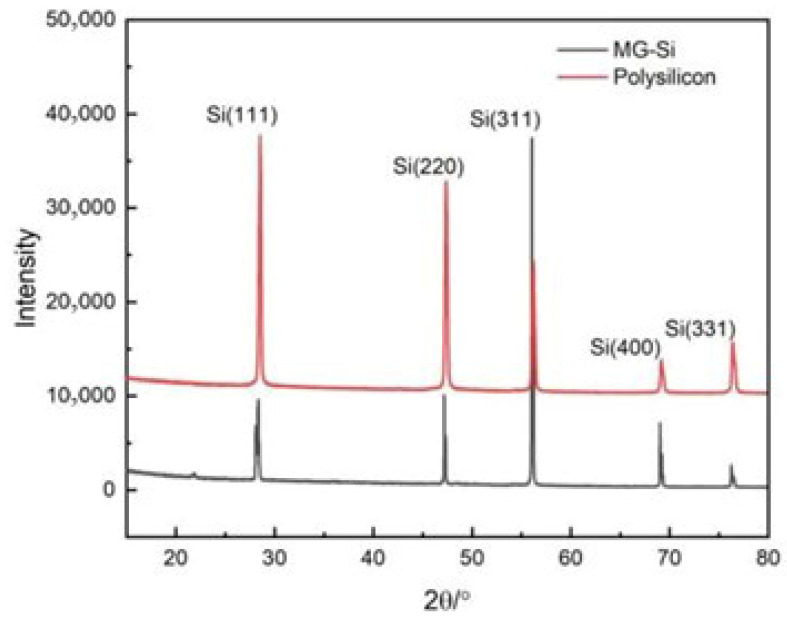
Phase composition of metallurgical silicon and polysilicon.

**Figure 3:**
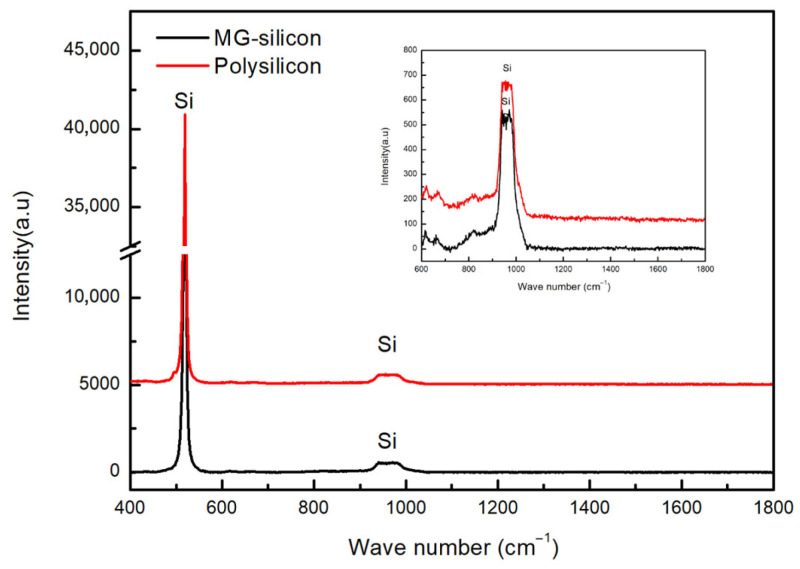
Raman spectra of metallurgical silicon and polysilicon.

Literature studies have indicated that metallurgical silicon is one of the primary source of carbon impurities in high-purity electronic-grade polysilicon [6]. Therefore, in this work, X-ray diffraction (XRD) technology was employed to characterize the phase compositions of metallurgical silicon and polysilicon powder, aiming to determine the existence forms of carbon impurities, as illustrated in Figure 2. Characteristic diffraction peaks associated with silicon (ICCD PDF-4 + 04-001-7247) were detected in the XRD patterns of both metallurgical silicon powder and polysilicon powder. The 2θ values at 28.4°, 47.3°, 56.1°, 69.2° and 76.4° correspond to the (111), (220), (311), (400) and (331) crystal planes of elemental silicon, respectively. In terms of phase composition, no obvious difference was observed between the two powders, except for the intensity variations in the diffraction peaks from different crystal planes. No characteristic peaks related to carbon-containing impurity phases were identified, which might be ascribed to the detection limit of the XRD method. Figure 3 shows the Raman spectra of metallurgical silicon and polysilicon, The characteristic peaks at wavenumbers of 520 cm^−1^ and 960 cm^−1^ are associated with the fundamental and overtone frequencies of the stretching vibration of the Si-Si bond, respectively. Similar to XRD, no characteristic peaks associated with C (1350 cm^−1^ and 1580 cm^−1^) and SiC (777 cm^−1^, 799 cm^−1^, 802 cm^−1^ and 905 cm^−1^) were observed in the Raman spectra of metallurgical silicon and polycrystalline silicon.

In this work, six metallurgical silicon samples and six polysilicon powder samples were selected, and their carbon contents were determined using a carbon-sulfur analyzer. The results showed that the carbon impurity content in the six metallurgical silicon samples was 0.012~0.023%, while the carbon impurity contents in the six polysilicon powder samples were all below the detection limit of the method (0.01%) and thus not detected. Furthermore, the floating zone (FZ) crystal growth method combined with cryogenic infrared spectroscopy was employed to measure the carbon impurity content in the six polysilicon samples. The results indicated that the carbon impurity content in the six single crystal silicon samples was 22~36 ppb (Table 1). Compared with the minimum concentration threshold for silicon carbide formation at the melting point of silicon (approximately 65 ppm; exceeding this value results in the formation of silicon carbide compounds in the melt) [25,26], the carbon impurity concentration in metallurgical silicon powder (denoted as C_MG−si_) is higher, while the carbon impurity concentration in high-purity polysilicon (denoted as C_EG−si_) is lower. In other words, the minimum concentration threshold for the formation of silicon carbide compounds in the silicon melt (C_mc_, 65 ppm) lies between the carbon impurity concentrations of metallurgical silicon powder and polysilicon (C_MG−si_ > C_mc_ > C_EG−si_). Therefore, in metallurgical silicon powder, the main existing forms of carbon impurities are a mixture of dissolved carbon atoms and silicon carbide, whereas in polysilicon, carbon impurities mainly exist in the form of dissolved atoms.

### 3.3. Evolution of Carbon Impurities and HSC Simulation of Reaction Thermodynamics

SiHCl_3_ is the main raw material for producing polysilicon. It is mainly prepared by cold hydrogenation technology, with the rest supplemented by direct synthesis. Therefore, the main sources of carbon impurities in SiHCl_3_ include metallurgical silicon, hydrogen gas, and SiCl_4_. According to the literature report during the processes of direct synthesis and cold hydrogenation reactions, silicon carbide in metallurgical silicon has no reaction activity, and the substances that participate in the reaction are the dissolved carbon atoms in metallurgical silicon [27]. The reaction system is mainly composed of elemental silicon, dichlorosilane (SiH_2_Cl_2_), SiHCl_3_, SiCl_4_, hydrogen, HCl, and corresponding impurities. SiH_2_Cl_2_, SiHCl_3_, SiCl_4_, hydrogen and HCl undergo cleavage, generating free radicals such as SiCl_2_·, SiCl_3_·, Cl·, H·, SiHCl_2_·, and SiHCl·. Subsequently, the free radical rearrangement occurs, producing the reaction products (SiH_2_Cl_2_ and SiHCl_3_) [10,28]. During this process, the solid solution carbon atoms in the metallurgical silicon react with hydrogen to produce CH_4_. A portion of the CH_4_ undergoes cracking to generate CH_3_· free radicals. Additionally, the methylchlorosilane compounds in chlorosilane and the CH_4_ in the recycled hydrogen also crack, leading to the formation of CH_3_· free radicals. The CH_3_· free radicals combine with Cl· free radicals to form chloromethane (CH_3_Cl), which then reacts with elemental silicon to generate methylchlorosilanes in different forms (Rochow reaction). Meanwhile, the methyl radicals (CH_3_·) combine with silicon-containing radicals including SiCl_2_·, SiCl_3_·, SiHCl_2_·, SiHCl· and hydrogen radicals (H·), forming a mixture of methylchlorosilanes and CH_4_ [10,29].

**Figure 4:**
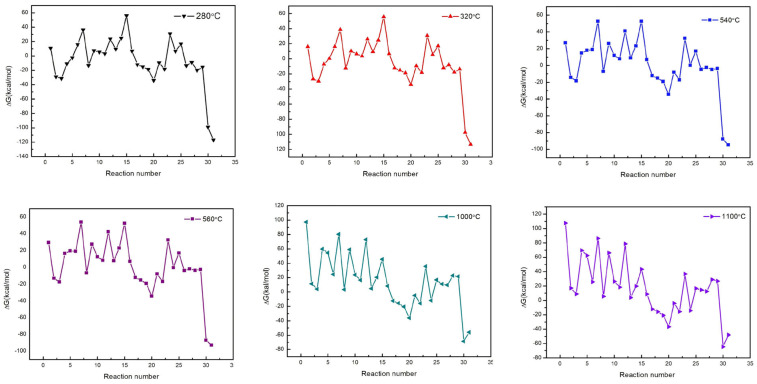
Gibbs free energy of reactions 1–31 at different temperatures.

The raw materials for the direct chlorination process are metallurgical silicon and HCl. The chlorine-containing substances in the reaction system include HCl, SiHCl_3_, SiH_2_Cl_2_, SiCl_4_, as well as the chlorides generated by the reaction between impurities in metallurgical silicon and chlorine-containing substances. Under certain conditions, carbon impurities in the metallurgical silicon can be converted into carbon-containing organic impurities, such as SiH(CH_3_)Cl_2_, Si(CH_3_)_3_Cl, Si(CH_3_)Cl_3_, Si(CH_3_)_2_Cl_2_, and undetectable organosilanes. Gibbs free energy is a thermodynamic parameter for evaluating the direction and extent of chemical reactions, and it is a state function. Its value is only related to the initial and final states of reactants and products, rather than the process path. Therefore, this paper uses the Gibbs free energy of the overall reaction to determine spontaneity. The possible reactions of carbon impurities and their corresponding Gibbs free energy values are shown in Table 2 and Figure 4. It can be seen that in the absence of a chlorine source, the Gibbs free energy of the reaction between carbon, silicon and hydrogen is positive. This means the reaction is non-spontaneous, and no Si(CH_3_)_4_ is generated in the system. When HCl is used as the chlorine source, silicon, carbon, hydrogen, and HCl react to form a silicone mixture. Among these reactions, the Gibbs free energy for the formation of Si(C_6_H_5_)Cl_3_ is nearly zero, resulting in a low reaction driving force. All other reactions proceed spontaneously, specifically, the reactions producing Si(CH_3_)Cl_3_ and SiH(CH_3_)Cl_2_ have lower Gibbs free energy and thus higher reaction driving forces. The chemical reaction between SiCl_4_, hydrogen, and carbon is non-spontaneous, which is similar to that of SiHCl_3_. The reaction between SiH_2_Cl_2_, carbon, and hydrogen produces SiH(CH_3_)Cl_2_, but does not generate Si(CH_3_)_3_Cl. The reactions between CH_4_, and SiHCl_3_ as well as SiCl_4_ are non-spontaneous. Si(CH_3_)Cl_3_ reacts spontaneously with hydrogen to produce CH_4_ and SiHCl_3_, while the reaction between SiH(CH_3_)Cl_2_ and hydrogen is non-spontaneous. Si(CH_3_)Cl_3_ reacts spontaneously with both SiHCl_3_ and SiH_2_Cl_2_, and all reactions produce SiH(CH_3_)Cl_2_. SiH(CH_3_)Cl_2_ undergoes a spontaneous reaction with HCl, producing CH_4_ and SiHCl_3_. Si(CH_3_)Cl_3_ can undergo a decomposition reaction to form Si(CH_3_)_3_Cl and SiCl_4_. However, the decomposition reactions of SiH(CH_3_)Cl_2_ and CH_4_ are non-spontaneous. In the direct chlorination reaction system, the order of Gibbs free energy for spontaneous reactions is ΔG_20_ < ΔG_3_ < ΔG_2_ < ΔG_19_ < ΔG_22_ < ΔG_18_ < ΔG_8_ < ΔG_17_ < ΔG_4_ < ΔG_21_. Among reactions of the same type, the Gibbs free energy for the reaction generating SiH(CH_3_)Cl_2_ is lower. Additionally, the reaction of Si(CH_3_)Cl_3_ with chlorosilanes to form SiH(CH_3_)Cl_2_ is spontaneous, which indicates that SiH(CH_3_)Cl_2_ is more likely to form. Although the Gibbs free energy of Reaction 22 is relatively low, the content of SiCl_4_ is much higher than that of Si(CH_3_)Cl_3_; therefore, the concentration of Si(CH_3_)_3_Cl is relatively low. The Gibbs free energy of Reaction 25 is positive, indicating that polyatomic hydrocarbon compounds such as ethane cannot exist stably in this system. The Gibbs free energy of Reactions 26 to 29 is negative, meaning that methane and elemental carbon are more prone to form under hydrogen-rich conditions when carbon monoxide and carbon dioxide are present in hydrogen gas. In summary, in the direct chlorination reaction system, the main existing forms of carbon impurities are SiH(CH_3_)Cl_2_ and CH_4_, accompanied by trace amounts of Si(CH_3_)_3_Cl and Si(CH_3_)Cl_3_.

Since the material system involved in the cold hydrogenation reaction is basically similar to that of direct chlorination, with only differences in the proportions of SiHCl_3_ and SiCl_4_ and in the reaction temperature, the spontaneity of the relevant reactions is similar to that of direct chlorination. The difference, however, is that the Gibbs free energy of Reaction 4 changes from a negative value to a positive value, and the order of the magnitudes of the Gibbs free energy for spontaneous reactions becomes ΔG_20_ < ΔG_22_ < ΔG_19_ < ΔG_3_ < ΔG_18_ < ΔG_17_ < ΔG_8_ < ΔG_2_ < ΔG_21_. This results in the product composition and the main existing forms of carbon impurities being similar to those in the direct chlorination reaction.

The distillation process mainly focuses on the separation of the main components (SiH_2_Cl_2_, SiHCl_3_ and SiCl_4_) and the purification of SiHCl_3_. Meanwhile, it is accompanied by the redistribution and enrichment of methylchlorosilanes in different components, but does not involve the conversion between different compounds. Since the boiling point of SiH(CH_3_)Cl_2_ (41–42 °C) is close to that of SiHCl_3_ (31.2 °C), and the boiling point of SiCl_4_ (57.6 °C) lies between those of SiH(CH_3_)Cl_2_ and Si(CH_3_)Cl_3_ (66 °C), after rough distillation separation, SiH(CH_3_)Cl_2_ is enriched in SiHCl_3_ (at the top of the tower), while Si(CH_3_)Cl_3_ is enriched in SiCl_4_ (at the bottom of the tower); in addition, the bottom product also contains a small amount of SiH(CH_3_)Cl_2_. After multiple rounds of distillation purification, SiH(CH_3_)Cl_2_ becomes the main impurity in the refined SiHCl_3_. Since the boiling point of CH_4_ is much lower than that of chlorosilanes, CH_4_ enters the hydrogen recovery system.

In the reduction process, the main raw materials are high-purity SiHCl_3_ (containing a small amount of SiH_2_Cl_2_) and hydrogen. The products include HCl, Si, SiCl_4_, SiH_2_Cl_2_, along with unreacted hydrogen and SiHCl_3_. That is, the main material system is Si-Cl-H, which is similar to the cold hydrogenation reaction. The main existing forms of carbon impurities in the raw materials are SiH(CH_3_)Cl_2_ and CH_4_. Concurrent with the reactions of the main material system, free radical generation and rearrangement reactions similar to those occurring in the cold hydrogenation process also take place, producing CH_4_ and methylchlorosilanes. Meanwhile, CH_4_ cracking reactions occur, which generate methyl free radicals and elemental carbon [30,31]. The reactions between carbon impurities and the main material system, along with their Gibbs free energy, are shown in Table 3.

Carbon (graphite) is one of the important sources of carbon impurities in polysilicon. Carbon atoms diffuse from graphite electrodes into the root of polysilicon rods, thereby forming carbon-headed silicon [3,6,7]. At 1000~1100 °C, in the coexistence of silicon and carbon, the Gibbs free energy of the reactions between them with H_2_, HCl, and chlorosilanes to form methylsilane, methylchlorosilanes, and phenylchlorosilanes is positive. These forward reactions are non-spontaneous, indicating that methylchlorosilanes are unstable in the reduction system and prone to decomposing into elemental carbon. The Gibbs free energy of the reactions between chlorosilane and CH_4_ as well as between SiH(CH_3_)Cl_2_ and hydrogen is positive, indicating that these reactions are non-spontaneous in the forward direction. In contrast, the Gibbs free energy values for the reactions of Si(CH_3_)Cl_3_ with hydrogen and of SiH(CH_3_)Cl_2_ with HCl to form CH_4_ are negative, meaning these reactions proceed spontaneously forward. Additionally, the reactions between Si(CH_3_)Cl_3_ and chlorosilane to generate SiH(CH_3_)Cl_2_ also exhibit negative Gibbs free energy, confirming their spontaneity. These thermodynamic characteristics provide favorable conditions for the existence of CH_4_ and SiH(CH_3_)Cl_2_ in the system. The Gibbs free energy for the decomposition of Si(CH_3_)Cl_3_ to form Si(CH_3_)_3_Cl is negative, whereas the Gibbs free energy for the decomposition of SiH(CH_3_)Cl_2_ to form Si(CH_3_)_3_Cl is positive. Combined with the high concentration of SiCl_4_ in the system, these results indicate that trace amounts of Si(CH_3_)_3_Cl may exist in the system. The Gibbs free energy of all reactions leading to the formation of Si(CH_3_)Cl_3_ is positive, indicating that Si(CH_3_)Cl_3_ cannot exist stably in the system. Under hydrogen-rich conditions, the Gibbs free energy for the formation of ethane (alkane, Reaction 25) via methane decomposition is positive, indicating that no other hydrocarbon compounds apart from methane are generated in the reaction system. It can be concluded from the Gibbs free energy of the reaction that CH_4_ tends to decompose to generate elemental carbon in the reduction system. Given the low concentrations of CH_4_ impurities in high-purity hydrogen and SiH(CH_3_)Cl_2_ impurities in SiHCl_3_, and considering that the solubility of carbon in silicon melt is approximately 65 ppm, carbon exists in high-purity polysilicon in the form of solute atoms. Unlike the direct chlorination and cold hydrogenation systems, the Gibbs free energy values of the reduction reactions of carbon monoxide and carbon dioxide (Reactions 26 to 29) are dominant in the CVD system. However, the Gibbs free energy values of Reactions 30 and 31 are negative, meaning that carbon monoxide and carbon dioxide tend to be reduced by elemental silicon to form silicon dioxide and elemental carbon. In other words, carbon monoxide and carbon dioxide cannot exist stably in this system. In summary, the main existing forms of carbon-related impurities in the reduction system are solute carbon atoms, CH_4_, and SiH(CH_3_)Cl_2_, accompanied by a small amount of Si(CH_3_)_3_Cl.

When producing high-purity polysilicon using the improved Siemens process, the recovered hydrogen mainly consists of two parts: reduction-recovered hydrogen and hydrogenation-recovered hydrogen. After separation and purification treatment, the reduction-recovered hydrogen is fed into the CVD system for recycling, with the regenerated hydrogen entering the cold hydrogenation system. The hydrogenation-recovered hydrogen is fed into the cold hydrogenation system for recycling, with the regenerated hydrogen discharged as waste gas. In the condensation and absorption/desorption process (tail gas recovery process), hydrogen in the tail gas is separated from chlorosilanes and HCl. High-boiling compounds such as chlorosilanes and methylchlorosilanes are fed into the subsequent distillation system, while HCl is absorbed by chlorosilanes. Low-boiling-point compounds such as hydrogen are introduced into the reduction or cold hydrogenation system. Compared with methylchlorosilanes, CH_4_ has a lower boiling point (−161.5 °C), and thus it enters the reduction and cold hydrogenation units along with hydrogen. In contrast, methylchlorosilanes follow chlorosilanes into the distillation process for further separation. Consequently, the carbon impurities in the recovered hydrogen are mainly in the form of CH_4_.

### 3.4. Sampling and Analysis Results of Carbon-Containing Materials at Different Sites

To verify the reliability of the above simulation results, this work conducted test and analysis on chlorosilane and hydrogen at key positions, in addition to metallurgical silicon and high-purity polysilicon. Among the results, Table 3 presents the analysis results of carbon impurities in crude chlorosilane generated by the synthesis process. It can be seen from the table that for the crude chlorosilane from the direct chlorination process (with a SiHCl_3_ content of ≥85%), the carbon impurities are mainly composed of CH_3_SiHCl_2_, with a small amount of Si(CH_3_)Cl_3_, while (CH_3_)_3_SiCl and (CH_3_)_2_SiCl_2_ are not detected. Before distillation separation, in the chlorosilane generated by the cold hydrogenation process (with a SiHCl_3_ content of approximately 27–29%), the carbon impurities mainly exist in the form of CH_3_SiHCl_2_ and Si(CH_3_)Cl_3_. (CH_3_)_3_SiCl and (CH_3_)_2_SiCl_2_ are not detected (Table 3). In contrast to the crude trichlorosilane (SiHCl_3_) produced in the direct chlorination synthesis process, the product herein has a higher content of Si(CH_3_)Cl_3_, which is mainly attributed to the high concentration of Si(CH_3_)Cl_3_ in the silicon SiCl_4_ feedstock.

Whether by direct chlorination or cold hydrogenation process, it is necessary to subject the chlorosilane mixture to crude distillation and separation treatment. The overhead product of the crude distillation column is further purified to obtain high-purity refined SiHCl_3_, which is then fed into the reduction system, while the bottom product SiCl_4_ is sent to the cold hydrogenation system for recovery and reuse. This study conducted a carbon impurity test and analysis on the purified SiHCl_3_ entering the reduction process, and the results are shown in Table 4. In the purified SiHCl_3_, carbon impurities mainly exist in the form of CH_3_SiHCl_2_, and other carbon-containing compounds are not detected. This study also tested the carbon impurities in the bottom product (mainly composed of SiCl_4_) after the crude separation of chlorosilane produced by cold hydrogenation, and the results are shown in Table 4. In the chlorosilane at the bottom of the crude distillation column, carbon impurities are mainly composed of Si(CH_3_)Cl_3_, with a small amount of CH_3_SiHCl_2_ also present.

In this work, 10 samples of reduction-recovered hydrogen and 10 samples of hydrogenation-recovered hydrogen were selected, and their carbon impurity compositions were tested. The results are shown in Table 5; the carbon impurities in the two types of recovered hydrogen mainly exist in the form of CH_4_. No other forms of carbon-containing compounds are detected, and the CH_4_ content in the hydrogenation-recovered hydrogen is higher. In conclusion, after separation treatment via rectification and tail gas recovery processes, carbon impurities mainly exist in the forms of CH_3_SiHCl_2_, Si(CH_3_)Cl_3_ and CH_4_ in SiHCl_3_, SiCl_4_ and recovered hydrogen, respectively, which is consistent with the results of HSC simulation calculations.

**Figure 5:**
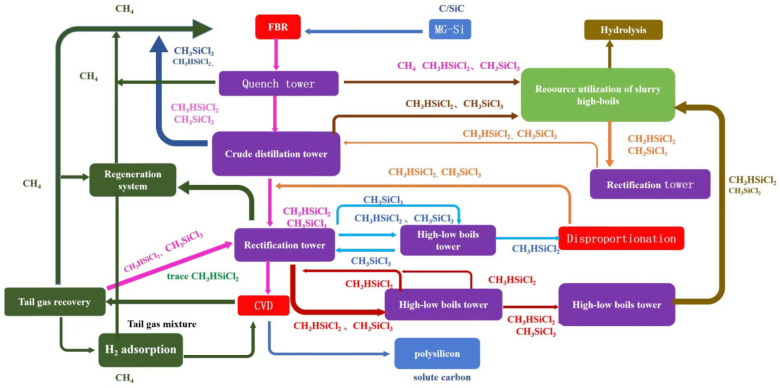
Evolution diagram of carbon impurities in the polysilicon preparation process via the modified Siemens process.

Combining the results of HSC simulation calculations and detection analysis, the evolution process of carbon impurities during the production of polysilicon via the improved Siemens process is as follows (see Figure 5): a mixture of solid-dissolved carbon atoms and silicon carbide (in metallurgical silicon), SiH(CH_3_)Cl_2_ (in SiHCl_3_) and Si(CH_3_)Cl_3_ (in SiCl_4_), solid-dissolved carbon atoms (in polysilicon) and CH_4_ (in recycled hydrogen).

The evolution mechanism of carbon impurities is that the solid-dissolved carbon atoms in metallurgical silicon react with hydrogen to generate CH_4_. Subsequently, CH_4_ undergoes a cracking reaction synergistically with methylchlorosilane compounds in chlorosilane and CH_4_ in recovered hydrogen, yielding CH_3_· radicals. The CH_3_· radicals combine with Cl· radicals to form chloromethane (CH_3_Cl), which then reacts with elemental silicon via the Rochow reaction. Meanwhile, the CH_3_· radicals combine with radicals including SiCl_2_·, SiCl_3_·, SiHCl_2_·, SiHCl· and hydrogen radicals (H·), forming a mixture of methylchlorosilanes dominated by SiH(CH_3_)Cl_2_ as well as CH_4_. After tail gas separation and distillation purification, SiH(CH_3_)Cl_2_ enters the SiHCl_3_ stream and is subsequently fed into the reduction process. In the reduction process, it undergoes cracking synergistically with CH_4_ in high-purity hydrogen to generate elemental carbon, which deposits in polysilicon in the form of substitutional atoms. Simultaneously, radical rearrangement reactions occur, producing a mixture of methylchlorosilanes dominated by SiH(CH_3_)Cl_2_ along with CH_4_, which are then discharged into the tail gas system. Therefore, in the production process of high-purity polysilicon, focus should be placed on developing efficient removal strategies for SiH(CH_3_)Cl_2_ and CH_4_ (see Figure 3).

## 4. Conclusions

Based on HSC simulation calculations and the Gibbs free energy of reactions, this study investigates the existing forms and evolution mechanism of carbon impurities during the polysilicon production via the improved Siemens process. The main conclusions are as follows:

(1) The minimum concentration threshold for the formation of silicon carbide (SiC) compounds in the silicon melt (65 ppm) is higher than the carbon impurity concentration in polysilicon but lower than that in metallurgical silicon. The main existing form of carbon impurities in metallurgical silicon is a mixture of solute carbon atoms and SiC. In contrast, carbon impurities in high-purity polysilicon mainly exist in the form of solute atoms.

(2) In the direct synthesis and cold hydrogenation processes, the main potential existing forms of carbon impurities are SiH(CH_3_)Cl_2_ and CH_4_, accompanied by the by-production of Si(CH_3_)_3_Cl and Si(CH_3_)Cl_3_. Through distillation purification, redistribution and enrichment of methylchlorosilanes occur among different components. Among these components, the carbon impurities in refined SiHCl_3_ are mainly present in the form of SiH(CH_3_)Cl_2_.

(3) In the reduction system, the main existing forms associated with carbon impurities are substitutional carbon atoms, CH_4_ and SiH(CH_3_)Cl_2_, accompanied by a small amount of Si(CH_3_)_3_Cl. These substances enter the tail gas recovery system, and after gas–liquid separation, CH_4_ accounts for the main component in the recovered hydrogen.

(4) In the production process of polysilicon via the improved Siemens method, the evolution process of carbon impurities is as follows: a mixture of solid-dissolved carbon atoms and silicon carbide (metallurgical silicon), SiH(CH_3_)Cl_2_ (in SiHCl_3_) and Si(CH_3_)Cl_3_ (in SiCl_4_), solid-dissolved carbon atoms (in polysilicon) and methane (in recycled hydrogen). This process is basically consistent with the sampling and testing results of different working procedures.

## Figures and Tables

**Table 1:** Carbon impurity concentrations in metallurgical-grade silicon and polysilicon.

Metallurgical-Grade Silicon	Sample	1	2	3	4	5	6
	Concentration (%)	0.015	0.023	0.012	0.018	0.015	0.021
Polysilicon	Sample	1	2	3	4	5	6
	Concentration (ppb)	26	36	29	34	30	22

**Table 2:** Possible reactions of carbon impurities and their Gibbs free energy during the direct chlorination process.

No.	Reaction Equation	ΔG (kcal/mol)						
		25 °C	280 °C	320 °C	540 °C	560 °C	1000 °C	1100 °C
1	Si + 4C + 6H_2_(g) = Si(CH_3_)_4_(g)	−35.39	10.53	16.05	26.91	29.44	97.24	107.47
2	Si + C + 3HCl(g) = Si(CH_3_)Cl_3_(g)	−43.54	−29.10	−26.82	−14.34	−13.20	11.53	17.11
3	Si + C + 2HCl(g) + H_2_(g) = SiH(CH_3_)Cl_2_(g)	−44.03	−31.51	−29.50	−18.46	−17.45	4.16	8.92
4	Si + 3C + HCl(g) + 4H_2_(g) = Si(CH_3_)_3_Cl(g)	−35.28	−10.99	−7.07	14.68	16.67	59.87	69.46
5	Si + 6C + 3HCl(g) + H_2_(g) = Si(C_6_H_5_)Cl_3_(g)	−22.75	−2.63	0.55	18.09	19.69	54.47	62.45
6	SiCl_4_(g) + C + 3H_2_(g) = SiH(CH_3_)Cl_2_(g) + 2HCl(g)	13.61	15.87	16.31	18.95	19.20	24.69	25.84
7	SiCl_4_(g) + 3C + 6H_2_(g) = Si(CH_3_)_3_Cl(g) + 3HCl(g)	22.37	36.40	38.80	52.55	53.84	80.40	86.36
8	SiH_2_Cl_2_(g) + C + H_2_(g) = SiH(CH_3_)Cl_2_(g)	−19.10	−13.29	−12.32	−7.13	−6.69	3.44	5.61
9	SiH_2_Cl_2_(g) + 3C + 4H_2_(g) = Si(CH_3_)_3_Cl(g) + HCl(g)	−10.35	7.26	10.11	26.01	27.47	59.15	66.14
10	SiHCl_3_(g) + C + H_2_(g) = Si(CH_3_)Cl_3_(g)	−0.77	5.45	6.45	12.03	12.54	23.87	26.45
11	SiHCl_3_(g) + C + 2H_2_(g) = SiH(CH_3_)Cl_2_(g) + HCl(g)	−1.26	3.04	3.78	7.91	8.29	16.51	18.42
12	SiHCl_3_(g) + 3C + 5H_2_(g) = Si(CH_3_)_3_Cl(g) + 2HCl(g)	7.49	23.56	26.21	41.05	42.42	72.88	78.8
13	SiHCl_3_(g) + CH_4_(g)= SiH(CH_3_)Cl_2_(g) + HCl(g)	10.81	9.59	9.38	8.90	7.97	4.80	3.94
14	SiCl_4_(g) + CH_4_(g) = Si(CH_3_)Cl_3_(g) + HCl(g)	26.18	24.82	24.58	23.26	23.13	20.36	19.72
15	SiCl_4_(g) + 3CH_4_(g) = Si(CH_3_)_3_Cl(g) + 3HCl(g)	58.60	56.04	55.56	52.65	52.37	45.30	43.43
16	SiH(CH_3_)Cl_2_(g) + H_2_(g) = CH_4_(g) + SiH_2_Cl_2_(g)	7.03	6.71	6.71	6.94	6.98	8.26	8.71
17	Si(CH_3_)Cl_3_(g) + H_2_(g) = CH_4_(g) + SiHCl_3_(g)	−11.31	−12.00	−12.05	−12.22	−12.23	−12.17	−12.14
18	Si(CH_3_)Cl_3_(g) + SiHCl_3_(g) = SiH(CH_3_)Cl_2_(g) + SiCl_4_(g)	−15.37	−15.22	−15.21	−15.16	−15.16	−15.56	−15.78
19	Si(CH_3_)Cl_3_(g) + SiH_2_Cl_2_(g) = SiH(CH_3_)Cl_2_(g) + SiHCl_3_(g)	−18.33	−18.71	−18.77	−19.16	−19.20	−20.43	−20.85
20	2Si(CH_3_)Cl_3_(g) + SiH_2_Cl_2_(g) = 2SiH(CH_3_)Cl_2_(g) + SiCl_4_(g)	−33.71	−33.93	−33.98	−34.32	−34.36	−35.98	−36.62
21	SiH(CH_3_)Cl_2_(g) + HCl(g) = CH_4_(g) + SiHCl_3_(g)	−10.81	−9.60	−9.38	−8.09	−7.97	−4.84	−3.94
22	3Si(CH_3_)Cl_3_(g) = Si(CH_3_)_3_Cl(g) + 2SiCl_4_(g)	−19.96	−18.42	−18.21	−17.12	−17.03	−15.76	−15.73
23	3SiH(CH_3_)Cl_2_(g) = Si(CH_3_)_3_Cl(g) + SiHCl_3_(g) + SiH_2_Cl_2_(g)	29.12	30.73	30.98	32.37	32.50	35.76	36.67
24	CH_4_(g) = C + 2H_2_(g)	12.08	6.55	5.60	0.18	−0.32	−11.70	−14.31
25	2CH_4_(g) = C_2_H_6_(g) + H_2_(g)	16.30	16.81	16.86	16.95	16.95	16.80	16.75
26	CO(g) + H_2_(g) = C + H_2_O(g)	−21.85	−13.42	−12.07	−4.59	−3.90	11.16	14.57
27	CO_2_(g) + 2H_2_(g) = C + 2H_2_O(g)	−15.01	−9.03	−8.04	−2.43	−1.93	9.82	12.54
28	CO(g) + 3H_2_(g) = CH_4_(g) + H_2_O(g)	−33.92	−19.97	−17.68	−4.78	−3.59	22.86	28.90
29	CO_2_(g) + 4H_2_(g) = CH_4_(g) + 2H_2_O(g)	−27.08	−15.58	−13.65	−3.65	−2.62	21.52	26.86
30	CO_2_(g) + Si = C + SiO_2_	−110.44	−99.15	−97.39	−87.85	−86.99	−68.61	−64.52
31	2CO(g) + Si = 2C + SiO_2_	−139.12	−116.96	−113.50	−94.59	−92.89	−56.11	−47.90

**Table 3:** Detection results of carbon impurities in crude chlorosilane produced by the direct chlorination process and cold hydrogenation process (ppm).

Sample No	CH_3_SiHCl_2_		CH_3_SiCl_3_		(CH_3_)_3_SiCl		(CH_3_)_2_SiCl_2_	
	DC	HC	DC	HC	DC	HC	DC	HC
1	1.02 × 10^3^	1.65	121	30.8	/	/	/	/
2	1.03 × 10^3^	1.18	131	242	/	/	/	/
3	1.17 × 10^3^	1.40	192	176	/	/	/	/
4	1.14 × 10^3^	1.33	150	83.1	/	/	/	/
5	1.17 × 10^3^	2.52	82.3	24.5	/	/	/	/
6	901	3.22	71.2	47.5	/	/	/	/
7	927	14.5	87.7	255	/	/	/	/
8	1.09 × 10^3^	8.14	92.5	2.04	/	/	/	/
9	1.39 × 10^3^	6.04	121	107	/	/	/	/
10	1.36 × 10^3^	5.31	44.4	84.5	/	/	/	/

Note: “/” indicates not detected, meaning the concentration is below the detection limit (1 ppm), DC and HC represent direct chlorination and cold hydrogenation, respectively.

**Table 4:** Analysis results of carbon impurities in purified trichlorosilane and chlorosilane at the bottom of the crude distillation column (ppm).

Sample No	CH_3_SiHCl_2_		CH_3_SiCl_3_		(CH_3_)_3_SiCl		(CH_3_)_2_SiCl_2_	
	Purified TCS	STC	Purified TCS	STC	Purified TCS	STC	Purified TCS	STC
1	7.22	/	/	195	/	/	/	/
2	9.03	1.17	/	544	/	/	/	/
3	8.21	/	/	214	/	/	/	/
4	2.82	/	/	254	/	/	/	/
5	5.84	1.73	/	649	/	/	/	/
6	3.18	/	/	175	/	/	/	/
7	7.87	1.48	/	766	/	/	/	/
8	3.51	1.22	/	735	/	/	/	/
9	5.03	1.07	/	699	/	/	/	/
10	3.91	1.54	/	764	/	/	/	/

Note: Purified TCS and STC represent direct chlorination and chlorosilane at the bottom of the crude distillation column, respectively.

**Table 5:** Detection results of carbon impurities in two types of recovered hydrogen (ppm).

Sample No	Reduction-Recovered Hydrogen				Hydrogenation-Recovered Hydrogen			
	CO	CO_2_	CH_4_	Methylchlorosilane	CO	CO_2_	CH_4_	Methylchlorosilane
1	/	/	0.92	/	/	/	371	/
2	/	/	1.17	/	/	/	339	/
3	/	/	0.84	/	/	/	397	/
4	/	/	1.08	/	/	/	381	/
5	/	/	1.08	/	/	/	318	/
6	/	/	0.93	/	/	/	337	/
7	/	/	0.70	/	/	/	341	/
8	/	/	0.95	/	/	/	319	/
9	/	/	0.60	/	/	/	329	/
10	/	/	0.66	/	/	/	329	/

Note: Methylchlorosilanes are mainly composed of CH_3_SiHCl_2_, Si(CH_3_)Cl_3_, (CH_3_)_3_SiCl, and (CH_3_)_2_SiCl_2_.

## Data Availability

The original contributions presented in this study are included in the article. Further inquiries can be directed to the corresponding author.
